# Epigenetic modulation in sensitizing metastatic sarcomas to therapies and overcoming resistance

**DOI:** 10.20517/cdr.2021.88

**Published:** 2022-01-04

**Authors:** Jeff Rytlewski, Qierra R. Brockman, Rebecca D. Dodd, Mohammed Milhem, Varun Monga

**Affiliations:** ^1^Department of Internal Medicine, University of Iowa, Iowa City, IA 52242, USA.; ^2^Holden Comprehensive Cancer Center, University of Iowa, Iowa City, IA 52242, USA.

**Keywords:** Epigenetics, sarcoma, resistance, metastasis

## Abstract

Sarcomas are a class of rare malignancies of mesenchymal origin with a heterogeneous histological spectrum. They are classically associated with poor outcomes, especially once metastasized. A path to improving clinical outcomes may be made through modifying the epigenome, where a variety of sarcomas demonstrate changes that contribute to their oncogenic phenotypes. This Perspective article identifies and describes changes in the sarcoma genome, while discussing specific epigenetic changes and their effect on clinical outcomes. Clinical attempts at modulating epigenetics in sarcoma are reviewed, as well as potential implications of these studies. Epigenetic targets to reverse and delay chemotherapy resistance are discussed. Future directions with primary next steps are proposed to invigorate the current understanding of epigenetic biomarkers to enact targeted therapies to epigenetic phenotypes of sarcoma subtypes. Modifications to prior studies, as well as proposed clinical steps, are also addressed.

## INTRODUCTION

Transitioning cancer from a lethal disease to a chronic one necessitates long-term limiting of cancer growth. Underpinning mechanisms of growth and proliferation are classically thought of as activation of an oncogene or the inhibition of a former tumor suppressor^[1]^. Some well-known examples of oncogenic driver mutations include EGFR-activation in non-small cell lung cancer and HER2 receptor amplification in breast cancer^[2,3]^. Pharmacological advances have led to the ability to target some of these drivers of tumor growth, resulting in significant clinical benefits. The development of imatinib is perhaps one of the best examples of this effect. After targeting the pathophysiologic driver BCR-ABL in chronic myeloid leukemia, complete response improved from 14.5% to 76.2%^[4,5]^. Imatinib is used in the management of gastrointestinal stromal tumors to target KIT or PDGFRα gain of function mutations which is present in the majority of tumors^[6]^. Imatinib has been reported to have a transformative effect on the median overall survival in patients with metastatic disease from months to years^[7,8]^. The possibility exists that some oncogene targets may be “undruggable” according to experts in the field, naturally leading to where the next best step may lie^[9]^. Mechanisms of resistance often develop to druggable targets via changes in drug metabolism, cell repair, and epigenetic effects^[10]^. Immunotherapy is emerging as a potential treatment option; however, response varies significantly depending on histological subtype and individual tumor pathology^[11,12]^.

Sarcomas are a family of malignancies mostly derived from cells of mesenchymal origin^[13]^. Composed of more than 50 different subtypes, they are differentiated between soft tissue *vs.* bone origin, and up to 50% of patients develop metastatic disease, where median survival is 10-15 months^[14]^. Treatment of sarcomas remains difficult as the variety of histological subtypes demonstrates varying sensitivities to chemotherapies^[15]^. If localized, surgical resection and perioperative radiation remain the hallmark of treatment, whereas anthracycline-based chemotherapy predominates treatment of advanced soft tissue sarcoma^[15-17]^. Like soft tissue sarcoma, management of localized osteosarcomas also relies heavily on wide local resection of the tumor with a preoperative and postoperative multi-drug chemotherapy regimen (high-dose methotrexate, doxorubicin, and cisplatin)^[18,19]^. Treatment algorithms to both osteosarcoma and soft tissue sarcoma have remained the same over the past several years, with minimal improvements driving a desire for innovation^[20,21]^.

Epigenetic modulation is a process linked to disease progression and therapeutic resistance (breast cancer, multiple myeloma)^[22]^. Dawson and Kouzarides^[23]^ describe epigenetics as “chromatin-based events that regulate DNA-templated processes”. Common mediators of epigenetics include histone de/acetylation and de/methylation, DNA de/methylation, and non-coding miRNAs that control mRNA translation without changing DNA cellular structure^[24]^. Cellular disruption of epigenetic control could be detrimental to long-term or induction treatment efficacy and is a postulated mechanism of resistance for various cancers^[25,26]^. One such example occurs in estrogen receptor-positive breast cancer, where the developed loss of *ARID1A* expression reduces the ability of estrogen to bind and thus decreases the therapeutic efficacy of selective estrogen receptor modulators^[27]^. Similar resistance effects have been reported when hypermethylation of DNA decreased the efficacy of cisplatin therapy in non-small cell lung cancer^[28]^.

## EPIGENETIC BACKGROUND OF SARCOMA

### Soft tissue sarcomas

Epigenetic changes have been implicated in the proliferation of multiple soft tissue sarcomas (STS) subtypes through a variety of different epigenetic mechanisms^[29]^. Common contributors to STS progression include TP53 (47%), CDKN2A (22%), retinoblastoma 1 (RB1; 22%), neurofibromin 1 (NF1; 11%), and ATP-dependent helicase (ATRX; 11%)^[30-32]^. While mutational profiles of these genes have been characterized, recent efforts have improved our understanding of epigenetic alterations of these genes and the correlation to STS progression and prognosis^[29-33]^. For example, mechanisms of CDKN2A inactivation include histone post-translation modifications by polycomb repressive complex 2 (PRC2), hypermethylation of DNA at promoter CpG sites, and loss of function (LOF) mutations^[32,34,35]^. There have been several studies, including a phase 2 clinical trial in liposarcoma and dedifferentiated liposarcoma, investigating the efficacy of cyclin-dependent kinase 4/6 inhibitors such as palbociclib in the setting of CDKN2A loss^[36,37]^. While the majority of STS with decreased CDKN2A expression exhibit LOF mutations in CDKN2A, loss of methylation in the CDKN2A promoter region has been shown to coincide with epigenetic transdifferentiation of uterine myosarcoma cells^[38]^. These findings suggest that hypomethylation in the CDKN2A promoter correlates with increased heterogeneity with hypomethylated tumors containing liposarcomatous and/or rhabdomyosarcomatous components^[38]^. Further investigation of DNA methylation in myosarcoma and other sarcomas could highlight novel mechanisms of tumor heterogeneity which have proven to be challenging to manage. Inhibition of other cell cycle players and their epigenetic modulators, such as the PRC2 and G2-checkpoint kinase WEE1, has also shown some preclinical efficacy in rhabdomyosarcoma (RMS)^[39-41]^. A recent publication by Zoroddu *et al.*^[42]^ discussed the essential role of PRC2 in muscle cell differentiation, a key component of RMS progression, and a potential therapeutic avenue for RMS patients. Furthermore, targeting the catalytic subunit of PRC2, enhancer of zeste homolog 1/2 (EZH1/2), should be further explored in combination with inhibitors that target the cell cycle kinases CDK4/6 and Wee1.

Targeting epigenetic modulators at the DNA and histone levels has been reported to show efficacy in altering specific pathways implicated in STS pathogenesis as well as sensitizing sarcomas to radiation and chemotherapies. For example, synovial sarcoma (SS) is characterized by a chromosomal translocation t(X; 18)(p11.2; q11.2) that frequently results in an SS18-SSX fusion protein^[43]^. This fusion includes a subunit of BRG1/BRM associated factor, ATP-dependent chromatin remodel complex, and SS-associated t(X; 18) genes, respectively. This fusion has also been reported to alter epigenetic histone modulators [SWItch/Sucrose Non-Fermentable (SWI/SNF) complex, Polycomb Repressive Complex 1 (PRC1), and Polycomb Repressive Complex 2 (PRC2) complexes], ultimately resulting in hypermethylation and subsequent gene silencing^[43]^. Additionally, other sarcomas characterized by chromosomal translocations exhibit a similar effect on PRC2 and SWI/SNF, as mentioned, as well as histone deacetylases (HDAC)^[44]^. HDAC activity has been implicated in sarcoma pathogenesis and is associated with advanced disease and poor clinical outcomes^[45]^. For example, HDAC activity is shown to play a role in immunoreactivity (HDAC 1, 4, 6-8) in endometrial stromal sarcoma^[45]^. The use of HDAC inhibitors is a promising therapeutic option. HDAC inhibition has been shown to inhibit growth while sensitizing tumors to chemotherapy^[45]^. While a better understanding of chromosomal translocation-dependent epigenetic rewiring is needed, treatment of SS tumors could benefit from the use of epigenetic therapeutic agents like EZH1/2 and HDAC inhibitors.

### Bone sarcomas

Investigation of epigenetics in bone sarcoma pathogenesis and potential treatment modalities has mainly focused on epigenetic mechanisms such as DNA methylation and histone modifications independently; however, mechanistic crosstalk between DNA methylation and histone modifications has been further elucidated in osteosarcomas and chondrosarcomas. A study by Li *et al.*^[46]^ observed that treating osteosarcoma cells with 5-aza-2’-deoxycytidine (5-Aza-CdR, decitabine) enhanced radiosensitivity by inducing a G2/M checkpoint arrest and induced apoptosis. These studies alluded to 5-Aza-CdR induction of DNA damage sensitizing the osteosarcoma cells to radiation. Furthermore, a common epigenetic marker for DNA damage is γH2AX, a double-stranded break DNA damage-induced histone modification^[47]^. Future studies characterizing γH2AX as a biomarker for new treatment modalities such as DNA methylation inhibitors in combination with radiation therapy could be beneficial for stratifying which radiation-resistant tumors could be re-sensitized using this approach.

Chondrosarcoma (CS) is another bone sarcoma subtype for which DNA and histone methylation correlate and represent multiple angles to treating tumor resistance and recurrence. Mutations in isocitrate dehydrogenase, an enzyme involved in α-ketoglutarate (α-KG) production in the Krebs cycle, have been documented in 52%-59% of central and 57% of dedifferentiated CS^[48,49]^. These mutations result in the production of an oncometabolite, δ-2-hydroxyglutarate, which is linked to a hypermethylated profile in CS tumors at the DNA/histone level, as well as promotion of malignant transformation^[48]^. Inhibiting both DNA methylation and histone methylation might be a useful approach to inhibiting the downstream function of this oncometabolite, known epigenetic compensatory mechanisms, and malignant transformation^[48]^. This concept has moved forward to clinical studies, where one such trial is evaluating the combination of HDAC inhibitor (belinostat) in combination with the hypomethylating agent guadecitabine (NCT04340843).

Ewing sarcoma is associated with a fusion oncoprotein Ewing Sarcoma breakpoint region 1-Friend leukemia integration 1 transcription factor (EWS-FLI1) that has been shown to be associated with epigenetic modulation, specifically DNA methylation, as well as histone acetylation and deacetylation^[49,50]^. Subsequent inhibition with sodium butyrate was also shown to be synergistic with vincristine, etoposide, and doxorubicin when grown *in vitro*^[51]^. Targeted inhibition of histone deacetylase likewise leads to a decrease in *in vitro *growth, reinforcing the potential promise of epigenetics in this specific cancer subtype^[52]^.

### DNA methylation-based classification of sarcoma

Sarcomas are a heterogeneous group stratified into many subtypes according to WHO criteria, which include distinct morphologies, immunohistochemistry, and disease-defining molecular events^[53]^. However, many subtypes lack unequivocal molecular hallmarks, resulting in discrepancies between diagnosis and treatment^[53-56]^. To help decrease these discrepancies, epigenetic changes such as DNA methylation could represent a resource to better distinguish sarcoma subtypes^[39,57]^. One study by Koelsche *et al.*^[33] ^explored global DNA methylation levels using a machine learning algorithm to compare the methylome classification to initial diagnosis and using a publicly-available sarcoma classifier. Prototypical soft tissue and bone tumors were analyzed using either HumanMethylation450K BeadChip or the EPIC array. Following DNA methylome analysis of 1077 tumor cases, representing 54 histological types, 62 methylation classes were identified following unsupervised hierarchical clustering and t-Distributed Stochastic Neighbor Embedding. Identified methylation classes were defined as methylation corresponding to WHO classification (48/62), methylation corresponding with a subgroup of a WHO entity (9/62), methylation corresponding with two distinct WHO entities (3/62), or methylation representing a novel entity (2/62). The first novel classification included SARC (RMS-like), which exhibited rhabdomyoblast-like cell morphology and DICER1 mutations. The second novel classification included SARC (MPNST-like), which presents similarly to MPNST but retains H3K27me3, an epigenetic modification that alters DNA packaging and is lost in the majority of MPNST. This study identified distinct subgroups using DNA methylation that correlates with another epigenetic mechanism, histone methylation (H3K27me3). MPNST biology is one example in which DNA methylation and histone modifications have been correlated; however, little is known about the mechanisms of epigenetic crosstalk in sarcoma pathogenesis^[58]^. Paraffin-embedded and formalin-fixed tissues can be analyzed via HumanMethylation450K BeadChip or the EPIC array. Raw data is then uploaded and analyzed using the sarcoma classifier. This publicly available resource and ongoing research in epigenetic mechanisms of STS pathogenesis could greatly benefit STS patients as new therapeutic options become available. Understanding epigenetic mutations and mechanisms as well as emerging/available clinical applications could enhance STS standard of care, including diagnosis, prognosis, and treatment efficacy.

### Additional preclinical considerations

While baseline epigenetic changes of STS and bone sarcoma have been more extensively studied, few preclinical models have been assessed with respect to sarcoma cells previously treated with standard-of-care regimens. However, this approach has shown promise in preclinical models of other cancers^[59]^.

One study of melanoma demonstrates how combining epigenetic modulators with chemotherapy could be exploited in sarcoma with regard to acquired resistance^[60]^. Zakharia *et al*.^[60]^ used a combination of the BRAF inhibitor vemurafenib with low-dose decitabine, a DNA methyltransferase inhibitor, in metastatic melanoma patients who had progressed on prior vemurafenib monotherapy. A fixed-dose of vemurafenib was combined with dose-escalated decitabine in a phase Ib trial design. Of the 14 patients enrolled in the trial, 3 achieved complete response, 3 achieved partial response, and 3 maintained stable disease. Clinical benefit rate was 79%, with no dose-limiting toxicities. Given the dramatic clinical responses seen in a refractory patient population, further preclinical studies were initiated to explore the effect. Decitabine was first confirmed to deplete DNA methyltransferase1 *in vitro *at concentrations below that needed to induce apoptotic effects. Separately, vemurafenib alone was able to inhibit cell growth until day 32, whereas the combined decitabine/vemurafenib regimen *in vitro *was able to maintain a cytostatic effect until day 90. Interestingly, cells in the combination treatment arm still demonstrated resistance to vemurafenib despite the prolonged stasis of cell growth in the combination treatment arm. Further analysis of patient genomics could shed light on whether non-responding patients had any shared features, though the structure of the study did not lend itself to outcome-driven analysis.

Previously performed clinical trials indicate that epigenetic modifiers within STS and bone sarcoma may demonstrate a similar effect. However, a critical area for growth is the creation of preclinical models that mimic treatment-experienced sarcoma models. Patients exposed to multiple lines of therapy in clinical settings do not have a corresponding or at least similarly derived cell line off of which treatments can be attempted. One could expect that epigenetic mechanisms similar to those seen in epithelial malignancies will have a better opportunity to be discovered and later reversed with the advent of better preclinical models^[27,28]^.

## PREVIOUS CLINICAL ATTEMPTS AT EPIGENETIC MODULATION

### Soft tissue sarcomas

Epigenetic modulators as monotherapy for sarcoma have been attempted with some success, a selected summary of which is included in Table 1. Perhaps the best known is that of tazemetostat, an EZH2 inhibitor approved in unresectable epithelioid sarcoma, the first FDA-approved epigenetic therapy in a solid tumor^[61]^. Chu *et al.*^[44]^ examined a pan-histone deacetylase inhibitor as monotherapy in 17 recurrent and metastatic soft tissue sarcoma patients. The best response in this study was stable disease in 8 patients, with no objective response seen. While limited in patient number, these two studies would suggest that unless the epigenetic target plays a role as an oncogenic driver, as in epithelioid sarcoma and EZH2 gain of function mutation, future efforts should focus on combination therapies with known cytotoxic agents as opposed to epigenetic targets as monotherapies^[62]^.

**Table 1:** A summary of the selected pharmaceutical trials highlighted by the epigenetic target and most prevalent sarcoma subtypes treated in the trial

**Pharmaceutical(s)**	**Epigenetic target(s)**	**Sarcoma subtype(s)**
Tazemetostat	EZH2	Epithelioid sarcoma
SB939	Histone deacetylase	Synovial sarcoma Myxoid liposarcoma Endometrial stromal Ewing sarcoma
Vorinostat	Histone deacetylase	Not specified
Valproate	Histone deacetylase	Uterine leiomyosarcoma Liposarcoma Angiosarcoma Epithelioid sarcoma
Decitabine	DNA methyltransferase	Leiomyosarcoma Chondrosarcoma Adenosarcoma Carcinosarcoma
Hydralazine Valproate	DNA methyltransferase Histone deacetylase	Not specified

Dembla *et al.*^[63] ^enrolled 44 total patients, 26 diagnosed with STS, in a study evaluating the effect of pazopanib combined with either a histone deacetylase or inhibitors of mTOR, Her2, or MEK. The study was designed as a retrospective review of patients with metastatic sarcoma previously treated with, ideally, anthracycline-based chemotherapy analogous to the PALETTE study^[64]^. Half of the patients received histone deacetylase inhibitors. Overall survival and progression-free survival of STS patients taking pazopanib with an epigenetic modifier were 38.7 weeks and 14.5 weeks, respectively. Genomic analysis via the Cancer Genome Atlas was performed as well, demonstrating mutations in 46% of the targeted epigenomic modifiers (HDAC, mTOR, Her2, MEK). Significant co-alterations were discovered between the Ras/Raf/MAPK pathway and HDAC alterations as well as between the ERBB pathway and HDAC alterations, though the specific alterations were not reported, and STS were not differentiated from bone sarcomas. Conclusions of this study are difficult to draw, in part due to incompatibility with the reference standard overall survival of the PALETTE study, even after selecting for STS as bone sarcoma. Given that some patients in this study had undergone prior VEGF treatment, acquired resistance could explain this effect. The genomic analysis lends an opportunity to evaluate whether a synergistic effect exists between addressing multiple epigenetic pathways at once, especially the MAPK pathway and HDAC, as they were co-altered at a 97% rate. If the drop in reference to overall survival and this effect were mediated via epigenetics, it would also imply that reversing the resistance could be difficult (provided this resistance seen was due to the attempted mechanisms). As such, combining epigenetic modulators at the advent of treatment could be beneficial.

We, the authors, evaluated 46 patients diagnosed with unresectable metastatic STS with a combination regimen of gemcitabine/docetaxel, combination chemotherapy, VEGF inhibition with bevacizumab, and valproate as an HDAC inhibitor^[65]^. Nearly 74% of patients had received prior chemotherapy; 30% of patients had received prior gemcitabine and docetaxel. Seventeen patients required dose reduction from the study regimen, with main adverse events being hepatotoxicity, neurotoxicity, and hypertension. The best response within the trial was a complete response in a case of epithelioid sarcoma, as well as 6 partial responses in other sarcoma subtypes. Interestingly, of patients who had previously received combination gemcitabine and docetaxel, 61% responded to the addition of valproate and bevacizumab regimen with either partial response or stable disease. Bevacizumab as monotherapy has a 13% reported response rate in STS^[66]^, and prior studies in treatment naïve metastatic STS patients demonstrated a 31% response rate to gemcitabine, docetaxel, and bevacizumab triple therapy^[67]^. With a response rate of 15%, of which 74% had previously undergone prior therapy, the rate of response in this trial is likely attributable to prior bevacizumab monotherapy in a majority of patients, reversal of chemotherapeutic resistance with bevacizumab, valproate or both, or effective valproate monotherapy.

Another phase 1b trial conducted by our group treated metastatic soft tissue and bone sarcoma with standard gemcitabine chemotherapy and the DNA hypomethylating agent decitabine^[68]^. Thirty-one patients (25 with STS) were enrolled, with 7 deemed non-evaluable; notably, 11 had received prior gemcitabine therapy with documented progression. Partial response was seen in 4 patients, with stable disease seen in 10, with a clinical benefit rate of 58%. Sarcoma subtypes with partial responses were leiomyosarcoma, adenosarcoma, and carcinosarcoma. These results combined with prior treatment with gemcitabine would imply that decitabine therapy was able to correct prior resistance to therapy.

An interesting, multifaceted approach in the setting of solid tumor treatment was performed by Bauman *et al.*^[69]^. From the perspective that aberrant DNA methylation is found in prior smokers, DNA methylation was used as a target in previously treated solid tumor patients. A combination regimen of hydralazine, demonstrated to be a demethylating agent *in vitro*, as well as valproate, demonstrated to have histone deacetylation activity, was utilized prior to chemotherapy in a steady state^[70,71]^. Patients were monitored for dose-limiting toxicities, and the regimen was well-tolerated, with no grade 4 side effects reported, and 5 total grade 3 side effects among 27 patients. Notable responses were seen in 2 of 3 STS patients who maintained stable disease for 3 and 4 months on second-line treatment. Further survival data of sarcoma patients was not reported. While the STS sample size was small, the data suggest a therapeutic benefit with combination epigenetic therapy that would be well-tolerated. Chavez-Blanco *et al.*^[72]^ discovered a similar potentiation effect with xenograft fibrosarcoma cells in a murine model, revealing a higher antineoplastic effect of chemotherapy regardless of the mechanism if co-administered with hydralazine and valproate. Further, xenograft tumors undergoing combination therapy did not regrow after the final dose of treatment, whereas tumors treated with adriamycin monotherapy did recur, reinforcing a possible increased efficacy of the coadministration regimen.

### Bone sarcomas

Chu *et al.*^[44] ^used the same pan-histone deacetylase inhibitor monotherapy within 5 EWS-translocation sarcomas, 1 of which was diagnosed as Ewing’s sarcoma, and 2 were diagnosed as chondrosarcoma. The patient in the study with Ewing’s sarcoma had progression of disease whereas best response with chondrosarcoma had stable disease; EWS-translocation sarcomas had the highest percent tumor change from baseline compared to all other sarcoma subtypes in the study^[44]^.

Dembla *et al.*^[63] ^examined the effect of pazopanib combined with primarily histone de-acetylators or inhibitors of mTOR, Her2, or MEK. Overall survival and progression-free survival of osteosarcoma patients taking pazopanib with an epigenetic modifier were 35.2 weeks and 8.9 weeks, respectively. While referencing the PALETTE study in design, the PALETTE study did not include bone sarcomas in its original analysis. This cohort does not match expected pazopanib monotherapy overall survival and progression-free survival^[73]^. While possibly this is due to a detrimental effect of combination therapy with epigenetic modifiers, a genomic analysis via the Cancer Genome Atlas divided by sarcoma subtype beginning with soft tissue sarcoma and bone sarcoma could demonstrate a difference between the two groups^[63]^.

### Ongoing and recruiting clinical trials

Of the trials listed in Table 2, one important step forward involves the use of epigenetic modulators and immunotherapy. This is being used with EZH2 inhibitors, PARP-inhibitors, and DNMT inhibitors, with room for expansion into other epigenetic targets, especially if the results prove to be promising.

**Table 2:** Ongoing clinical trials evaluating epigenetics in the setting of sarcoma as documented by clinicaltrials.gov with associated clinical trial numbers

**Clinical trial number**	**Tumor type(s)**	**Regimen**	**Epigenetic target**	**Study start date**	**Estimated end date**
NCT04648826	Sarcoma, melanoma, germ cell	Aerosolized azacytidine and bintrafusp alfa	DNA methyltransferase	Oct 2021	Dec 2029
NCT04705818	Sarcoma, colorectal cancer, pancreatic cancer	Tazematostat and durvalumab	EZH2	Jul 2021	Jul 2023
NCT03694262	Endometrial sarcoma	Bevacizumab, atezolizumab and rucaparib	PARP-inhibitor	Jul 2019	Jun 2026
NCT03919539	Osteosarcoma	Famitinib and camrelizumab	Biomarker evaluation	Dec 2019	Dec 2021
NCT04340843	Chondrosarcoma	Belinostat, guadecitabine	DNA methyltransferase, histone deacetylase	Jul 2020	Jan 2022

## FUTURE DIRECTIONS

While a substantive amount of information relating to the role of epigenetics in the oncogenesis of sarcoma has only recently been discovered, further exploration to best derive benefit is still needed. Specifically, determining the epigenetic profile of an individual patient specimen is needed. While this is already being pursued in part by NCT03919539 in the evaluation of osteosarcoma, we suggest similar clinical studies in STS to better correlate epigenetic profile with disease progression, treatment susceptibilities with the current standard of care, and ultimately the development of targeted epigenetic therapies. Epigenetic profiling has already been shown to lead to the improved diagnostic characterization of sarcoma^[33]^.

Some positive outcomes in prior studies suggest epigenetic therapies can delay resistance to already effective chemotherapies, as demonstrated by Zakharia *et al.*^[60] ^in the context of vemurafenib and melanoma, with preclinical correlates. To better elucidate these effects in sarcoma, we suggest a trial of patients with metastatic STS undergoing standard of care chemotherapy enrolled to receive concurrent epigenetic therapy to better potentially identify a prolonged duration of response. Currently, it also appears that epigenetic therapies are able to play a role in returning the effectiveness of a previously used regimen^[63,65]^.

None of the epigenetic therapies used are able to specifically target only epigenomic changes related to sarcoma pathogenesis, leading to a possible concern over lack of specificity. Fortunately, one consistent determination among the studies reported here is that combination epigenetic therapy inhibiting multiple targets has thus far been well-tolerated by patients. We suggest combining current standard of care regimens with multiple epigenetic therapies as opposed to the monotherapies more frequently attempted, as these will inhibit any compensatory epigenetic changes seen in monotherapies. This will likely be more beneficial in the event further identification of the epigenome is performed and concurrent mutations are identified, as shown by Dembla *et al.*^[63]^.

An additional approach, as seen in the NCT04648826 and NCT04705818 trials involving epigenetic targets and immunotherapy, would involve utilizing therapeutic options previously considered less effective with epigenetic targets to evaluate a change in efficacy. If effective, we hypothesize that the epigenetic target would likely unmask a therapeutic benefit similar to the re-sensitizing of chemotherapy, as shown by Monga *et al*.^[65]^, though if the therapeutic benefit is found, more mechanistic characterization would need to be determined. One potential modification would be to utilize combination epigenetic targets as mentioned above.

The preclinical setting, as alluded to above, also has exciting potential for growth. Arguably one of the most beneficial changes would be the development of reflective treatment-experienced preclinical cell lines for STS and bone sarcomas for both characterization of suspected epigenetic mutations seen after treatment as well as reversing acquired resistance. Finally, many targets identified in preclinical settings have yet to be truly evaluated in a clinical context to determine their efficacy, as shown by Ciarapica *et al.*^[40]^ who determined that inhibiting WEE1 was a promising effective therapy in high-risk RMS. This oncogenic pathway is shared by the PRC2 complex, leading to the possibility that a similar therapeutic effect may be found if the EZH2 inhibitor tazemetostat is used.

Multiple directions of study will lead to ample opportunity for growth in the study of epigenetics, as well as possibly defining the role that epigenetic modulation may play in the treatment of sarcoma.

## DECLARATIONS

### Availability of data and materials

Data sharing is not applicable to this article as no new data were created or analyzed in this study.

### Copyright

© The Author(s) 2022.
